# Nurturing future leaders for nature: the example of the UK's National Education Nature Park

**DOI:** 10.1098/rstb.2022.0330

**Published:** 2024-06-10

**Authors:** Jonny Hazell, Edward Clarke

**Affiliations:** The Royal Society, London, London SW1Y 5AG, UK

**Keywords:** nature, children, biodiversity, schools, government, education

## Abstract

This piece reviews the evidence on the effect that engaging with nature has on school-age children's future attitudes and behaviour towards nature. It also looks into the impact engaging with nature has on children and young people's physical, mental and personal development and the evidence on how much time children in England are spending in natural environments. It then presents a UK Government project, the National Education Nature Park (NENP), intended to increase children in England's access to nature by developing nature spaces within the grounds of educational institutions. It concludes with suggestions for how the implementation of the NENP could be used to learn more about the effect that engaging with nature has on children alongside understanding how school-based nature projects could benefit the local environment.

This article is part of the theme issue ‘Bringing nature into decision-making’.

## 1. Impact of childhood experiences on concern for nature

As many societies become more urbanized and digitized, with a higher proportion of people living in towns and cities than in rural environments and spending more time on electronic devices, interactions with the natural world have declined [1]. This has raised concern about possible feedback loops whereby less time spent in nature leads to lower concern for its protection and so higher nature loss, which in turn reduces opportunities for connecting with nature. Several literature reviews have concluded that childhood is a crucial period for developing a sense of connection with nature. In a review of the benefits of children's engagement with nature, Gill [2] concludes that there is robust evidence to support the assertion that more time spent in nature as a child is associated with pro-environmental attitudes and feelings of being connected with the natural world as an adult. Similarly, Charles *et al.*'s [3] review concludes that positive, direct experiences of nature as a child and care for nature being modelled by someone close to the child are the two most important determinants of individuals choosing pro-environmental behaviours as an adult. More recently Chawla [4] found that people who spend more time in nature in their childhood are more likely to actively take care for nature in adulthood. Chawla [4] also concluded that children who report greater connection with nature are also more likely to report taking action to conserve nature.

Although many of the studies included in these reviews are dependent on correlational evidence and so have limited potential for quantifying the precise impact of childhood engagement with nature and adult attitudes and behaviours, together they indicate that increasing the time children spend in nature is important for developing a connection to nature, and this sense of connection is important for the likelihood of their adopting pro-environmental behaviours as adults. As will be discussed in greater detail below, opportunities to increase engagement with nature in urban environments are likely to be of greatest benefit in increasing overall engagement, and schools represent a significant opportunity in these urban environments. In terms of how these spaces are used to increase children's connection to nature, Gill [2] emphasizes the importance of ‘playful engagement styles' for developing a sense of connection with nature, whereas Sheldrake & Reiss [5] suggest that outdoor learning experiences and environmental education programmes can be important for fostering children's interest in learning about nature. This implies that nature spaces in schools should afford opportunities for both free play and be seen as a valuable learning resource.

As well as influencing future behaviour, there is a possibility that increasing children and young people's sense of connection to nature could have a more immediate effect. There is a consensus in business and academia that children have an increasing influence in the decision making process of families [6]. There is also strong support for the suggestion that environmental education can be transferred between generations and indirectly induce targeted behavioural changes [7]. Therefore it is possible that students' positive engagement with nature, coupled with high quality learning, could result in them influencing behaviour change at home which would have a positive impact on nature; for example by changing dietary habits or by making gardens more accommodating for nature.

## 2. Benefits of children's engagement with nature

As well as having benefits for future conservation, childhood engagement with nature is beneficial to children as well. The increasingly extensive research into the consequences of children engaging with nature has been reviewed in multiple meta-analyses [2,8–12]. These have established a broad-based body of evidence demonstrating an association between increased engagement with nature and improved physical and mental health and psychological development. The studies included in these reviews covered a wide range of ages (2–18 years old), environments (urban parks, gardens, playgrounds, farmland, woodland, coasts etc.), and activities (free play, formal/informal learning, gardening, conservation etc.). The extent to which engagement with nature leads to the benefits discussed below will be moderated by the age of the child, the type of environment, and the nature of the engagement. The National Education Nature Park (NENP) provides an opportunity to learn more about the benefits of the kinds of engagement afforded by spaces for nature in schools.

### (a) Physical health benefits

Numerous studies have found an association between greater nature engagement and higher physical activity [8,9]. As increasing physical activity is one of the World Health Organization's central recommendations for improving overall health [13], this is a positive outcome in itself, but there is correlational evidence of greater time spent in nature being associated with increased fitness in pre-school children [2] and reduced risk of being overweight or obese in children over the age of 6 years [10].

Although many of the physical health benefits of greater engagement with nature are likely to be mediated by increased physical activity, recent research has found that children who attend nature-oriented day-care centres in Finland have a more diverse microbiome than those attending urban day-care centres with little or no green space. A more diverse microbiome is associated with reduced risk of immune-mediated disease [14]. Crucially these researchers demonstrated that introducing natural elements—such as segments of forest floor, turf, planters for growing annuals, and peat blocks for climbing and digging—to these urban day-care centres led to microbiome changes in the children such that they became more like those of the children attending nature-oriented day-care centres.

### (b) Mental health benefits

An association between increased engagement with nature and improved mental health outcomes has also been established through multiple meta-analyses [8,9,15], although reviewers note methodological limitations in some of the studies, particularly a predominance of observational rather than experimental study designs, bias in the selection of study participants such that they are not representative of the target population, and limitations in the control of confounding variables. They therefore raise the possibility that higher quality research could lead to different conclusions. Research has looked at both impacts in the general under-18 years population and also particular sub-populations such as children with attention deficit disorder (ADD) and/or attention deficit hyperactivity disorder (ADHD). In the general under-18 years population, greater engagement with nature is associated with improved overall mental health as well as reduced stress and depression and increased resilience [1,2]. In children with ADD/ADHD, increased exposure to nature is associated with improvements in the symptoms of these disorders [2,9]. Specifically in the context of engagement with nature within school grounds, Harvey *et al.* [16] report that amongst 8–11 year olds who participated in a year-long programme to monitor biodiversity at their school, those that reported increased levels of nature connection at the end of the year also reported significant gains in wellbeing based on self-reported physical activity and health; general mood and feelings about self; friends, family and free time; and school and learning.

Whilst most studies into the association between nature and mental health have considered positive associations, Chawla [4] observes that many young people are struggling with the painful awareness of environmental degradation and instability. This emphasizes the importance of helping children to develop a sense of what Chawla describes as ‘constructive hope’—a sense of agency in what can otherwise feel like an overwhelmingly bleak environmental crisis. Chawla's review points to evidence that providing young people with opportunities to develop and deliver personally meaningful pro-environmental actions is an important element of a strategy to help them cope with environmental change. This research is important for ensuring that NENP projects are co-designed with school children and that children have an ongoing role in maintaining them. To achieve this, funding for NENP projects could be made contingent on demonstrating how school children were involved in project design.

### (c) Psychological development benefits

Mygind *et al.* [10] reviewed the literature on the extent to which children's social and emotional development are associated with engagement with nature. They found evidence of positive associations between natural environments and children's ability to form positive relationships, socially adaptive behaviours, emotion management and expression, and overall socioemotional adaptation. They speculate that these positive associations might be underpinned by improved working memory given the evidence that exposure to green space improves performance in tests of working memory.

Mann *et al.* [11] also found evidence of development of skills that support learning (concentration and engagement in particular) in a meta-analysis of the literature on nature specific outdoor learning. Across a range of interventions such as teaching lessons outdoors and school gardening programmes, they also found improvements in social skills such as teamwork, self-confidence and responsibility.

### (d) Educational outcomes

In addition to personal development, a number of meta analyses have found a correlation between increased engagement with nature and improved educational outcomes, although few of the studies that reached this conclusion were rated as high quality. In studies looking specifically at nature specific outdoor learning i.e. classes taught in a natural environment, Mann *et al.* [11] found good evidence of increased student engagement and a sense of greater ownership of their learning and some evidence of improved educational outcomes. Similar results were found by Becker *et al.* [12] (albeit from a lower number of studies), who attribute this outcome to an observed increase in motivation to learn. Scott *et al*. [17] hypothesize a complementary mechanism for how engagement with nature could lead to improved educational outcomes by supporting the development of language and communication skills.

As well as generalized benefits, there is evidence of subject-specific learning benefits from outdoor education including science [2] and natural history [5].

## 3. Levels of childhood engagement with nature in the UK

Given the evidence of the benefits of children engaging with nature, it is concerning that there is an apparent trend of children spending less time in natural environments. In England, government agency Natural England has been conducting an annual survey of children's (aged 15 or under) and young people's (aged 16–24) engagement with nature since 2009, although this was disrupted by the COVID-19 pandemic. The most up to date survey at the time of writing comes from data collected between March 2018 and February 2019. [18] This found that engagement with nature is falling for all children and especially for those from lower socioeconomic backgrounds. The survey also found that urban green spaces such as parks and playgrounds provide the greatest source of engagement for nature and that urban green spaces provided a disproportionately important opportunity for engaging with nature for children living in less affluent areas and those from racially minoritized communities. Taken together, these findings point to the potentially significant contribution that enhanced spaces for nature in school grounds could play for all children's experience of nature and especially for those living in less affluent areas and from racially minoritized communities.

## 4. Increasing children's access to nature at school: the National Education Nature Park

### (a) England's sustainability and climate change strategy

In 1992 the United Nations Framework Convention on Climate Change identified education as a priority area for action. However, it was not until 2022 that the Department for Education (DfE), who are responsible for education in England (the four nations of the UK—England, Northern Ireland, Scotland and Wales—are each responsible for their own education policy), launched its Sustainability and Climate Change Strategy (SCC).

The ambition is for ‘the United Kingdom to be the world-leading education sector in sustainability and climate change by 2030’ [19]. The strategy applies right across the education sector, from early years and schools (and independent schools where applicable) to further and higher education across England and describes five action areas:

(i) climate education: enhance and develop learning *about* the natural environment and learning *in* the natural environment, supporting teachers to do this;
(ii) green skills and careers: inspire more young people to choose career paths that support the transition to net zero, restoration of biodiversity and provide a sustainable future;
(iii) infrastructure and the education estate: make schools more sustainable by retrofitting schools, taking new approaches to sustainable building design, retrofit, information and communications technology, building management and the surrounding environment;
(iv) operations and supply chains: introduce sustainable business practises to schools; and
(v) international (collaborative working and trade opportunities): work across government to drive change across the UK nations as well as further afield.

The DfE hopes to achieve the strategy's aims through several initiatives such as the introduction of a new general certificate of secondary education in natural history, an awards scheme for schools, and the introduction of a new NENP [19].

### (b) The National Education Nature Park

English school grounds collectively cover 514 km^2^ [20]. By considering the whole physical education estate as a virtual NENP, the DfE [20] believes there is an opportunity to:

(i) deliver improvements in biodiversity;
(ii) contribute to the implementation of the nature recovery network;
(iii) play a part in halting nature's decline; and
(iv) drive greater climate resilience.

The project aims to give every young person in England opportunities to develop a meaningful connection to nature, understand the concepts of climate change and biodiversity loss and make them feel able to do something about it. Students across the country will be empowered to protect nature in their school grounds and create environments that improve biodiversity.

A coalition of organizations led by the UK's Natural History Museum are working to deliver this initiative [21]. Together they will work with schools to map, manage and enhance all the land across the education estate, creating a virtual nature park.

The NENP has been awarded £5.3 million of guaranteed funding for the first two years of the scheme and it will not be a statutory requirement for schools to engage with the scheme.

### (c) What will the National Education Nature Park involve?

The first stage will involve schools mapping their grounds' biodiversity so that gains in their area can be tracked. Esri UK, a well-known provider of digital mapping tools, will be devising tools for use by children and young people to enable them to map the biodiversity of their estate and its improvement over time.

Schools will then work to transform their green spaces into their own nature park. Students will play leadership roles in studying, managing and enhancing biodiversity and climate resilience in their nature park and local community. From creating pollinator-friendly habitats where biodiversity can thrive, to digging ponds or creating planting schemes that support climate resilience. The nature park will also give students the opportunity to take part in community science, and in biodiversity monitoring and data analysis.

Students will be supported to lead and manage their own nature park, modelling the staff of a national park or nature reserve as managers, ecologists, communicators, fundraisers, grounds people and data analysts. They should be able to choose, plan, and implement a range of site improvements and habitat enhancements based on the latest scientific evidence, and monitor biodiversity gains.

## 5. Likely impacts of the National Education Nature Park

The school estate in England (buildings and school land combined) covers an area twice the size of Birmingham (514 km^2^) [20,22]. If the entire school estate was given over to nature, it would make the NENP the size of a national park, greater in area than the Norfolk Broads (303 km^2^) and just shy of the New Forest (580 km^2^). However, the NENP will not receive the same protections that England's other national parks are given and schools will not be giving all their grounds over to nature. In fact the DfE is currently reviewing hundreds of school sites in a bid to find land suitable to be sold for housing [23]. Until the geospatial mapping exercise is complete, the actual size of the NENP will remain unknown.

**Figure 1.**
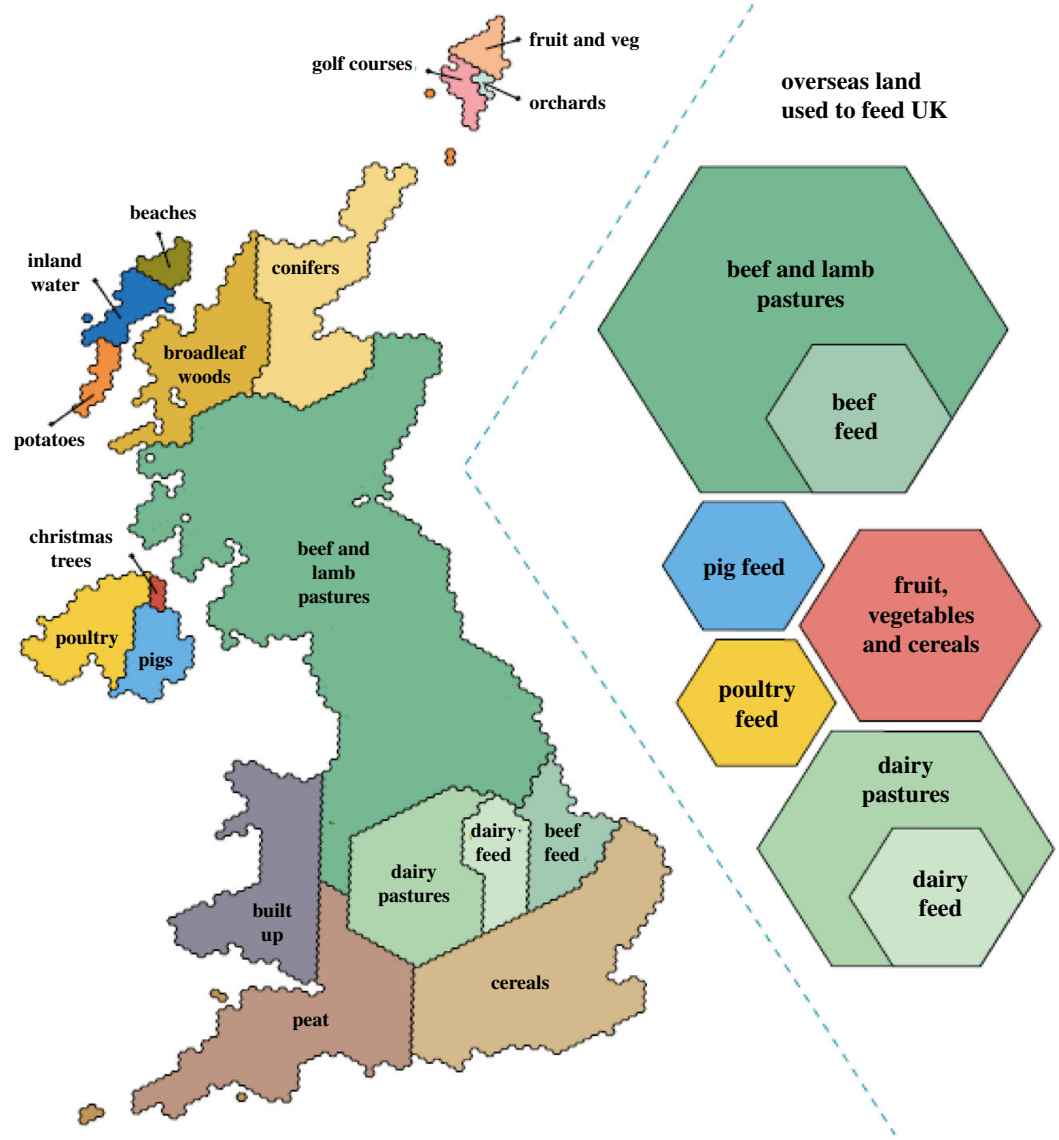
Land use in the UK (reproduced from the National Food Strategy 2021 [27,28]).

Compared to other uses of land across the UK, private residential gardens occupy a quarter of the area of a typical city (4523 km^2^) [24], while golf courses are estimated to take up between 227 km^2^ and 1522 km^2^ [25]. The majority of land in England is used for agriculture (89 000 km^2^) [26], the majority of which is used to farm sheep and cattle (figure 1). So the NENP is probably not large enough to have a significant impact on nature in England, especially when compared to other policy initiatives that shape how land is used.

However, school grounds could become ecological corridors and stepping stones to help species' long-distance dispersal and range expansion, especially when considering the fairly even distribution of schools across the country. This impact could be magnified if schools take a coordinated, local approach to improve regional biodiversity.

When £5.3 million of funding for the NENP (less than 0.01% of the schools budget) is divided between the 32 000 schools in England, each school will have about £165 to transform their grounds. This limited funding has been criticised as undermining the ability to deliver on the strategy. For example, Emma Knights, chief executive of the National Governance Association, responded: ‘There is much to welcome in the DfE strategy…But it seems unlikely that schools will be given the wherewithal to achieve what is rightly being asked of them’ [29].

### (a) Wider reception

Responses to the SCC and the concept of an NENP have been generally welcoming. However, many have noted the strategy does not go far enough or fast enough and falls short of the kind of serious reform that is required to address climate and ecological breakdown.

The National Association for Environmental Education noted that there are almost no measurable targets, and many of the commitments lack deadlines, meaning there is a risk the strategy will be delivered in a tokenistic or unimpactful way [30]. Meanwhile academics and union leaders have highlighted that in order to have a real impact, sustainability needs to be incorporated across the entire curriculum for students of all ages [31,32].

Indeed, the House of Commons Environmental Audit Committee recommended that the education system should be transformed so children from an early age to adulthood are encouraged to experience, celebrate, and learn about nature [33].

However, the Government has not signalled any intention to seriously reform education or the curriculum. In a House of Lords debate, Baroness Baron (a Minister at the DfE) stated the ‘[the Government] do not believe that amending the curriculum is the right way to encourage pupils to learn about a sustainable environment. The subjects of citizenship, science and geography all include content on sustainability and the environment, and schools have the autonomy to go into as much depth on these subjects as they see fit’ [34, column 1062].

## 6. Conclusion

Nature spaces in schools can help address the observed decline in children's engagement with nature. This is likely to be especially important for children living in urban areas from lower socioeconomic groups, helping them to access the benefits of engaging with nature. For all children, careful co-design of school nature projects could help develop a sense of ‘constructive hope’ in the face of anxiety related to environmental change. By also helping to develop a greater connection with nature, these projects can help inspire the next generation of business, industry and political leaders to act in the interests of nature when the responsibility is theirs.

A wealth of literature demonstrates the benefits engaging with nature can have for children. Given that reviews of this literature often highlight the mixed quality of this research, the NENP provides an opportunity to conduct some robust research to explore the mechanisms through which engagement with the kinds of spaces for nature that can be constructed within school grounds lead to positive outcomes. This research should inform the design of interventions that are tailored to deliver specific outcomes. It will also be important for this research to assess the benefits to nature of different project designs if these spaces are to be as useful to biodiversity as they are to children.

Myriad factors will determine the success of the SCC and the NENP. In the short term, its direct impact on nature is likely to be insignificant on national level, particularly without a longer term commitment and more significant funding, especially compared to other policy initiatives that address land use [35]. The challenge of funding for school-based projects raises the significance of improving the quality of nature in all spaces that children access as part of their education such as local parks.

Even if not significant in terms of direct impacts on nature, the NENP could be invaluable in getting young people engaged with and building a connection with nature which will have a lasting impact. If schools use it as a teaching opportunity, this could yield wider benefits as young people take their learning back home to help make communities and society more conscious of nature and its benefits.

For scientists, the NENP should be seen as a great opportunity for public engagement. Those based in England should be encouraged to engage with schools in their local area to see how they can help improve biodiversity, sustainability and promote science within their communities.

## Data Availability

This article has no additional data.
